# Judd–Ofelt Analysis and Spectroscopy Study of Tellurite Glasses Doped with Rare-Earth (Nd^3+^, Sm^3+^, Dy^3+^, and Er^3+^)

**DOI:** 10.3390/ma16216832

**Published:** 2023-10-24

**Authors:** Naziha Boudchicha, Mostepha Iezid, Faycal Goumeidane, Messaoud Legouera, P. Syam Prasad, P. Venkateswara Rao

**Affiliations:** 1 Applied Chemistry and Materials Technology Laboratory, Larbi Ben M’hidi University, Oum El Bouaghi 04000, Algeria; nazihaboudchicha@gmail.com; 2 Laboratoire d’Innovation en Construction, Eco-Conception et Genie Sismique (LICEGS), University Mostafa Ben Boulaid Batna 2, Route de Constantine, Fesdis, Batna 05078, Algeria; yazidmostefa@yahoo.fr; 3 Laboratory of Active Components and Materials, Larbi Ben M’hidi University, Oum El Bouaghi 04000, Algeria; 4 Laboratoire de Genie Mecanique et Matériaux, University 20 Aout 1955, Skikda 21000, Algeria; legouira@yahoo.fr; 5 Department of Physics, National Institute of Technology Warangal, Warangal 506004, Telangana, India; 6 Department of Physics, The University of the West Indies, Mona Campus, Kingston 7, Jamaica; pvrao54@gmail.com

**Keywords:** tellurite glasses, absorption, optical band gap, rare-earth, Raman spectroscopy, Judd–Ofelt

## Abstract

A series of glasses based on (80-y) TeO_2_-20 BiCl_3_-y RE_2_O_3_ (y = 0, 0.6 mol%; RE = Nd, Sm, Dy, and Er) were prepared. The thermal stability of the glass was determined by differential scanning calorimetry (DSC). The density and optical energy values of the prepared glass increased in the order of Sm_2_O_3_, Nd_2_O_3_, Dy_2_O_3_, and Er_2_O_3_. In addition, the glass doped with Er_2_O_3_ had the highest refractive index values compared to the other samples. Subsequently, Judd–Ofelt parameters (Ω_2_, Ω_4_, and Ω_6_) were obtained for the family of RE^3+^ trivalent rare-earth ions introduced as dopants in a tellurite glass. These parameters were calculated from the absorption spectra for each RE^3+^. The structures were studied by Raman spectroscopy deconvolution, which determined that TeO_4_, TeO_3_, TeO_3+1_, BiO_6_, and BiCl_6_ units had formed. In addition, the structural changes in the glass are related to the intensity ratio of TeO_4_/TeO_3_, depending on the type of rare-earth. For the optics and Judd–Ofelt parameters, the ray spectroscopy results of the prepared glass show that it is a good candidate for nonlinear optics fibers, a solid laser material.

## 1. Introduction

Nd, Sm, Dy, and Er are examples of rare-earth (RE) elements that have several multilateral uses in innovative technology [1]. Due to the fact that it is utilized to make optoelectronic components, including planar waveguides, optical detectors, fluorescent display technology, visible lasers, optical fibers, and optical amplifiers, rare-earth (RE) ion-doped glass has recently attracted interest [2].

Tellurite oxide-based glasses offer special physical characteristics features such as high linear and nonlinear indices of refraction, low melting points, simple forming and dimensions, high transparency, chemical resistance, high thermal stability, and infrared transmittance [3]. Glasses including TeO_2_ have potential applications in the fields of photonics; optoelectronics; and optical instrumentation, including laser technology, telecommunications, amplifiers, and electrical storage devices [4].

Additionally, tellurium dioxide is convertible with other glass materials, enabling the incorporation of several compositions inside tellurate glass. These glasses are thought to be effective hosts, enhancing the luminescence characteristics of a number of lanthanide ions. Therefore, it is interesting to peer into the tellurate glass’s structure, particularly when the glass is doped with rare-earth ions. An asymmetrical TeO_4_ trigonalbipyramidal (tbp), which contains two axials, two different kinds of sites, and three equatorial positions, is the distinctive structural unit of crystalline TeO_2_. An individual pair of electrons is present in one of the latter. There are two distinct types of fundamental structural units in TeO_2_-based glasses, namely TeO_4_ tbp and TeO_4_ tbp with a lone pair electron, which makes their structure interesting.

The addition of bismuth chloride (BiCl_3_) as a second glass forming helps in the creation of superior structural units, which have an impact on the physical and optical characteristics of the glass system. Tellurite glass research has attracted a lot of attention. Due to its potential applications in various industries, this has changed recently. Doped with appropriate rare-earth (RE) ions, tellurite glass is a candidate for use in optical fibers, nonlinear optics, lasers, solar cells, and sensors [5]. Sm_2_O_3_-doped glass exhibits and produces an intense orange-red luminescence in the visible region related to the ^4^G_5/2_ and ^6^H_9/2_ transitions, depending on the excitation wavelength. Glass that has Er_2_O_3_ is thought to be a potential material for optical amplifier production (EDFAS) and the application of optical communications.

The J–O intensity characteristics also reveal the glass bonding type, according to a number of scientists, in order to know more about the optical and physical characteristics of Nd^3+^, Sm^3+^, Dy^3+^, and Er^3+^ ions in the glass matrix, as well as the radiative lifetimes of erbium doped in tellurite glasses. Research has also looked into determining the feasibility of applying this glass system in optical glasses for photonics and lasers.

A reliable approach for determining estimated radiative properties such as the rate of spontaneous emission, duration, and branching ratio is the Judd–Ofelt analysis. Furthermore, the computation of the gain and quantum efficiency includes a few Judd–Ofelt spectroscopic parameters [6,7]. When choosing a vitreous matrix, several glasses’ chemical compositions have been adjusted. Low phonon energy considerably increases the probabilities of radiative transition in glasses such as fluoro-zirconate and heavy metal oxide-based glasses (HMOGs), making these glasses the best candidates for photoluminescence [8].

The current work uses the Judd–Ofelt method to investigate the radiative characteristics of tellurate glasses (TeO_2_-BiCl_3_) doped with lanthanide ions (Nd^3+^, Sm^3+^, Dy^3+^, and Er^3+^). In order to investigate whether these rare-earth ions influence the structural characteristics of the TB glass system, we provide general physical and optical characteristics. The structure of these glasses has been extensively studied using the Raman technique.

## 2. Materials and Methods

### 2.1. Glass Synthesis

**Figure 1:**
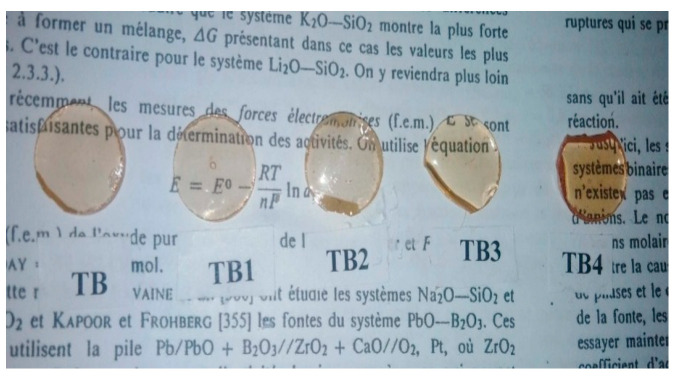
Photography of the samples.

With a chemical formula of (80-y) TeO_2_-20 BiCl_3_-y RE_2_O_3_ (y = 0, 0.6% mol; RE = Nd, Sm, Dy, and Er), glass samples from Sigma Aldrich (St. Louis, MO, USA) were created with a high purity of 99.9%. To remove adsorbed water, all chemical compositions were preheated in a furnace at 200 °C for 3 h. The glass sample code and material composition are shown in Table 1. Each of the components was weighed and combined in a mortar using a weight (about 5 g), and the mixtures were melted at a temperature of 750 °C in the air under flame heat for 10 min until a homogeneous liquid was obtained. Then, the melt was cast on a mold brass, maintained at a temperature lower than the glass transition (T ≈ T_g_ − 10 °C). The obtained glasses were annealed for 6 h at T_g_-20 °C in an electric furnace to eliminate the stresses induced during quenching. The photography of the samples is presented in the Figure 1. The samples were polished and cut to a few millimeters thick. The current study investigates the radiative properties of tellurate glasses (TeO_2_-BiCl_3_) doped with lanthanide ions (Nd^3+^, Sm^3+^, Dy^3+^, and Er^3+^) using the Judd–Ofelt method. We present general physical and optical features to evaluate whether these rare-earth ions affect the structural properties of the TB glass system. The Raman method has been used for considerable research on the structure of these glasses.

### 2.2. Characterization

By utilizing CuKα radiation at 40 kV and 100 mA during XRD studies SHIMADZU XRD 6000 (Chiyoda-ku, Tokyo, Japan) the materials’ amorphous natures are confirmed. The 2θ range had a step size of 0.04° per second and a range of 0–60°. Glass density measurements were taken with a precision of 0.001 g/cm^3^ utilizing the Archimedes method. The TA Instrument DSC Q20 model (New Castle, DE, USA) which has a greater sensitivity of 0.1 °C and a heating rate of 10 K/min, was used to record the samples’ thermal properties. The Tg value was judged to be 2 K, whereas the Tx and Tp values were 1 K. The Tx-Tg stability factor demonstrated the outstanding stability of our glasses. A 

Scienta Omicron R3000 spectrometer (Danmarksgatan 22, Uppsala, Sweden) operating between 100 and 1000 cm^−1^ was used for the Raman spectroscopy. Measurements of the optical transmissions in the UV–Visible band between 400 and 800 nm were performed using an Agilent Technologies Cary 5000 spectrometer (Santa Clara, CA, USA).

## 3. Results and Discussion

### 3.1. X-ray Diffraction Analysis

**Figure 2:**
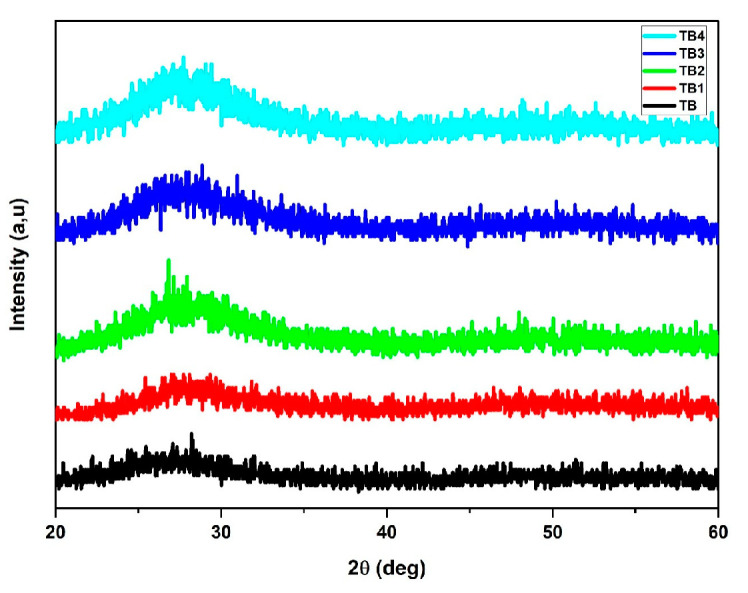
Typical XRD pattern of glass with the composition (80-y) TeO_2_-20 BiCl_3_-y RE_2_O_3_ (y = 0, 0.6% mol; RE = Nd, Sm, Dy, and Er).

The X-ray diffraction patterns of a system glass made of (80-y) TeO_2_-20 BiCl_3_-y RE_2_O_3_ (y = 0, 0.6% mol; RE = Nd, Sm, Dy, and Er) are depicted in Figure 2. A broad peak curve was seen between 20° and 30° of the 2θ. The absence of diffraction peaks confirms the amorphous structure of these glasses [9].

### 3.2. Thermal Properties

**Figure 3:**
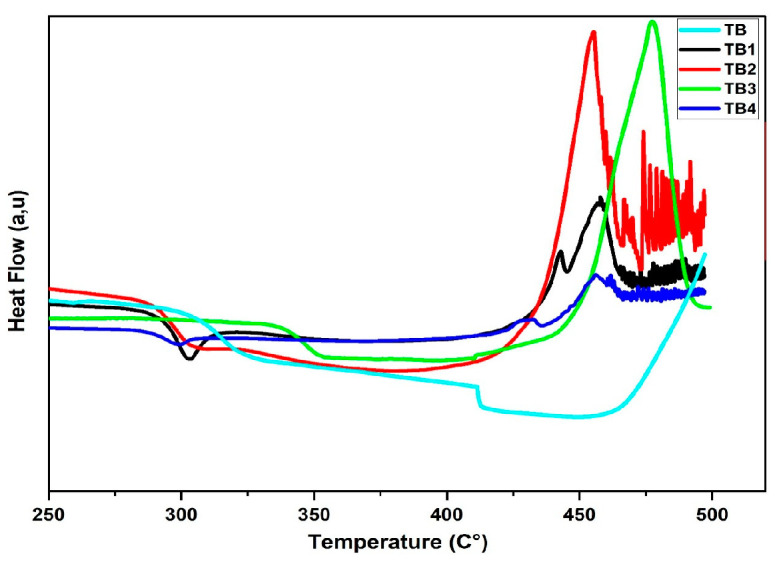
The DSC curves of the (80-y) TeO_2_-20 BiCl_3_-y RE_2_O_3_ (x = 0, 0.6% mol; RE = Nd, Sm, Dy, and Er) glass system.

Figure 3 displays the differential scanning calorimetry (DSC) patterns for each of the investigated glass samples: the glass transition temperature (T_g_), the onset crystallization peak temperature (T_x_), the peak of the crystallization temperature (T_p_), the thermal stability factor ΔT = T_x_ – T_g_ [10], and Hruby’s parameter H = (T_x_ – T_g_)/T_g_.

Table 2 lists the glasses’ thermal stability of anticrystallization or resistance to crystallization [11]. The T_g_, T_x_, T_p_, ΔT, H, and S are in the range of 285–333 °C, 390–439 °C, 430–477 °C, 116–127 °C, 0.32–0.44, and 7.54–15.63 °C, respectively, according to Table 2. The rigidity of the network is what causes an increase in the Tg with the addition of 0.6 mol% of Nd_2_O_3_, Sm_2_O_3_, Dy_2_O_3_, and Er_2_O_3_ in the glass system. Glass is considered thermally stable if the difference ∆T, which is used to calculate thermal stability, is larger than 100 °C. The ΔT was greater than 100 °C for all glass samples. TB4 had the highest glass stability (127 °C) [12].

Glass stability can be shown by Hruby’s parameters; a greater H denotes an improved degree of glass formation stability. The highest value of H is displayed by TB4 glass, with a value of 0.44 signifying the highest glass-forming potential. Because there is a significant correlation between the stability of the glass and exothermic broadness, the thermal stability, S, given by Saad and Bolin, stands out [13]. The resistance is reflected in the parameter S. The value at TB2 was the greatest, and the value at TB4 was the lowest. The alteration in solid-state bond formation in complex matrices is related to changes in the material’s thermal characteristics.

### 3.3. Physical, Optical Properties

The diverse physical parameters, including density (ρ_g_, g/cm^3^), the molecular weights (M_wt_, g/mol), molar volume (V_mol_, cm^3^/mol), oxygen volume (V_OXG_, cm^3^/mol), oxygen packing density (OPD, g atom/l), indirect optical band gap ($E_{opt}^{ind}$, eV), refractive index (n), molar refractivity (R_mol_, cm^3^/mol), molar polarizability (α_mol_, (A°)^3^), refraction loss (R_Loss_), dielectric constant (ε), polaron radius (r_pl_, A°), inter ionic distance (r_in_, A°), RE ion concentration (N × 10^20^, ions/cm^3^), and field strength (F_s_ × 10^14^, cm^−2^), were calculated using the expressions provided in the literature [14], and the estimated values are shown in Table 3. Equation (1) was used to calculate the density value (ρ_g_) [15].

(1) $$\rho g=\frac{u_{a}}{u_{a}-u_{b}}\rho_{\mathrm{wat}}$$

where *u_a_* and *u_b_* are the weights of the glass sample in air and in immersion liquid, respectively, and ρ_wat_ is the water density. The calculated density value of the prepared glass decreased from 5.506 g/cm^3^ to 5.468 g/cm^3^, which corresponded to TB and TB4 glass. Table 3 presents the results.

Rare-earth compounds are ranked according to their molecular weights (M_wt_) as follows: TB1 < TB2 < TB3 < TB4, with respective M_wt_ values of 191.879, 191.953, 192.098, and 192.156 g/mol. Erbium-doped glass is denser and more compact than undoped glass. Using Equations (2) and (3), find out how much glass there is in the sample and how much oxygen there is in the sample. Table 2 lists the density of the glass samples used to carry out these calculations.

(2) $$V_{\mathrm{mol}}=\sum \frac{X_{i}M_{i}}{\rho_{g}}$$

where *M_i_* is the molecular weight, and *X_i_* is the fraction ratio of every oxide. The oxygen molar volume, V_OXG_, can be calculated using the relationship below.

(3) $$V_{\mathrm{OXG}}=\left(\sum \frac{X_{i}M_{i}}{\rho_{g}}\right)\left(\frac{1}{\sum X_{i}n_{i}}\right)$$

where *n_i_* is the number of individual oxide oxygen atoms [11]. From 35.10 cm^3^/molto 35.01 cm^3^/mol, corresponding to TB1 and TB4, the molar volume, V_mol_, decreased. From 45.86 cm^3^/mol, V_O_ decreased to 45.74 cm^3^/mol. In addition, it is possible that the reduction in V_O_ reflected an increase in the synthesis of NBO atoms, given that the OPD was determined using Equation (4) [2].

(4) $$\mathrm{OPD}=1000\;\left(\frac{\rho_{g}\sum X_{i}n_{i}}{\sum X_{i}M_{i}}\right)$$

The value of OPD reduced from 21.87 to 21.65 g atom/l as the rare-earth ions in the glass matrix were doped.

The polaron radius r_pl_ (A°), inter ionic distance r_in_ (A°), and field strength Fs (×10^17^cm^−2^) were calculated from Equations (5)–(8) [15]. The concentration of ions:

(5) $$N_{\mathrm{RE}}=\frac{{RE}_{mol\%}\rho_{g}N_{AVG}}{M_{wt}}$$

(6) $$r_{\mathrm{pl}}\;(A{}^{\circ})={\frac{1}{2}(\frac{\pi }{6N_{i}})}^{\frac{1}{3}}$$

(7) $$r_{\mathrm{in}}\;(A{}^{\circ})={\left(\frac{1}{N_{i}}\right)}^{\frac{1}{3}}$$

(8) $$\mathrm{Fs}({\mathrm{cm}}^{-2})=\frac{A}{{r_{pl}}^{2}}$$

where (N_RE_) is the concentration of rare-earth ions, (N_AVG_) is the Avogadro’s number, (*ρ_g_*) is the density of the glass sample, A is the atomic mass of the rare-earth ions, (r_in_) is the mean distance between the RE^3+^ ions, (r_pl_) is the polaron radius, and (F_s_) is the field strength of TB glass that has been doped. Table 3 displays the results of the calculations. The estimated results show that, for RE^+3^ ions, ri and rp are identical. As a result, the field strength (F) surrounding Er^+3^ ions is enhanced, and the RE-O bond becomes more stable. When taken together with the intensity results, these values prove that the addition of Er^3+^ ions causes the glass structure to compress, which, in turn, reduces the degree of electron delocalization by increasing the number of donor centers in the glass matrix, which reduces the optical band gap [9].

Using Davis’s and Mott’s relation and Equation (9), the E*opt* for amorphous material was calculated [16].

(9) $$\left(\alpha h\upsilon \right)=Β{\left(h\upsilon -{Ε}_{opt}\right)}^{r}$$

**Figure 4:**
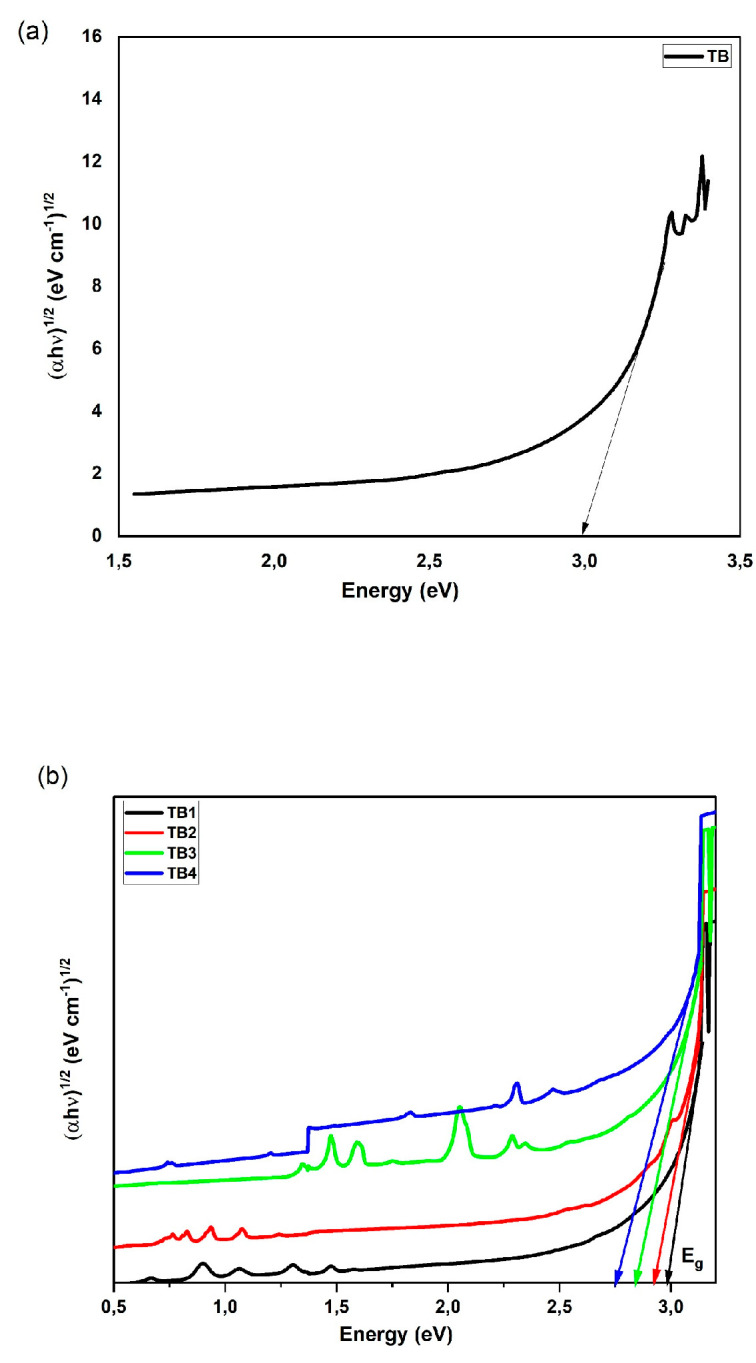
Plot of (αhʋ)^1/2^ against the energy of (**a**) a TB glass sample and (**b**) of doped glass samples for indirect band gap measurements.

For every value of r between 1/2 and 2, where *B* is a constant, allowable indirect transitions have an r value of 1/2, whereas direct transitions have an *r* value of 2 in Equation (10). The values of indirect E*opt* were calculated by extrapolating the linear fit on the x-axis of the plot of (αhν)^1/2^ against photon hν, as shown in Figure 4.

(10) (αhυ)12=Β(hυ−Εopt)

The E*opt* value ranges from 2.75 to 2.99 eV, depending on the kind of glass and how it was manufactured. Table 3 displays the values of E_opt_ for each sample, with TB having the highest value and TB4 the lowest.

The index of refraction n is one of the most important characteristics of optical glasses. The refractive index values of prepared glass samples were obtained using the Dimitrov and Sakka relation, as depicted in Equation (11) [17] and tabulated in Table 3.

(11) $$\frac{\left(n^{2}-1\right)}{\left(n^{2}+1\right)}=1-\sqrt{\frac{{Ε}_{opt}^{ind}}{20}}$$

With the change in the doping RE^3+^ ion concentration in the produced glass, the n value rises from 2.39 to 2.47. The molar mass, electron density, and ion polarity are all factors. Strong polarization of non-bridged oxygen production (NBO) in the glass lattice is responsible for the enhancement of the refractive index. The polarizability is increased due to the greater refractive index resulting from the creation of non-bridging oxygen when compared to covalent oxygen bridging bonds.

The calculation of the molar refraction (R_mol_, cm^3^/mol) is carried out by using the Lorentz–Lorenz Equation (12) [11]:

(12) $$R_{mol}=\frac{n^{2}-1}{n^{2}+1}\cdot V_{mol}$$

The calculation of the molar polarizability (α_mol_) can be determined using Equation (13):

(13) $$\alpha_{mol}=\frac{R_{mol}}{2.52}$$

Table 3 presents the values of polarizability (α_mol_) and molar refraction (R_mol_) for the glass samples doped with rare-earth ions. The dielectric constant (ε) and refraction loss (R_L_) are determined using Equations (14) and (15), respectively [11].

(14) $$\varepsilon =n^{2}$$

(15) $$R_{loss}={\left(\frac{n-1}{n+1}\right)}^{2}$$

The increase in the R_mol_, α_mol_, ε, and R_Loss_ can be attributed to an increase in the (NBO) and RE^3+^ ions in the glasses’ network structure.

### 3.4. Judd–Ofelt Analysis

Laser action and optical amplification are two phenomena for which the J–O theory is often utilized to make predictions. Bands of absorption were seen in this research, and it was shown that they were most often connected with transitions involving induced electric dipoles [7,18]. Oscillator strength was defined as the intensity of the absorption spectral lines. Furthermore, Equation (16) [8,14] was utilized to calculate the experimental oscillator strength ($f_{\exp}$) for each absorption band.

(16) $$f_{\exp}=4.32\times 10^{-9}\int \varepsilon \left(\upsilon \right)d\upsilon$$

where $\varepsilon \left(\upsilon \right)=\frac{1}{Ck}\cdot \log\frac{I_{0}}{I}$ represents the molar extinction coefficient in cm^−1^, *k* represents the optical path length in cm, *C* represents the concentration of RE^3+^ in mol.L^−1^, and ∫ε(υ)dυ represents the area under the absorption curve. The transition frequency ($f_{cal}$) between RE^3+^’s ground state (aJ) and excited state (aJ’) was calculated. Equation (17) was used to determine the ionization transition.

(17) $$f_{cal}=A_{ed}+A_{md}=\left[\frac{8\pi^{2}mc\upsilon }{3he^{2}\left(2J+1\right)n^{2}}\right]\left(X_{ed}S_{ed}+X_{md}S_{md}\right)$$

The variables $X_{ed}$ and $X_{md}$ represent Lorentz-localized field corrections that are accountable for the dipole transitions, and h is the Planck’s constant. They are written using the refractive index n as

(18) $$X_{ed}={\frac{\left(n^{3}+2n\right)}{9}}^{2}$$

(19) $$X_{md}=n^{3}$$

n is the glass sample’s determined refractive index. The line strength for both electric and magnetic dipoles, $S_{ed}$ and $S_{md}$, respectively, can be calculated as follows.

(20) $$S_{ed}\left(aj,aj^{\prime }\right)=e^{2}\sum_{\lambda =2,4,6}\Omega^{\lambda }{\left|\langle aj^{\prime }\rangle \right|}^{2}$$

(21) $$S_{md}=\frac{e^{2}}{4m^{2}c^{2}}{\left|\langle aJ^{\prime }\rangle \right|}^{2}$$

The values of the doubly reduced matrix elements of order Ω_λ_ (λ = 2, 4, 6) between the manifold states of ${\left|\langle aj^{\prime }\rangle \right|}^{2}$ and ${\left|\langle aJ^{\prime }\rangle \right|}^{2}$ were obtained from [14,19]. The J–O intensity parameters were calculated using the least squares fitting method. The root mean square (RMS) deviation was used to assess the quality of fitting between $f_{cal}$ and $f_{\exp}$.

(22) $$RΜSΕ={\left[\frac{\sum {\left(f_{cal}-f_{\exp}\right)}^{2}}{\varepsilon -3}\right]}^{2}$$

ε represents the total number of observed transitions. The probability of spontaneous transition $A\left(aJ,bJ^{\prime }\right)$ is given by

(23) $$A\left(aJ,bJ^{\prime }\right)=A_{ed}+A_{md}=\left[\frac{64\pi^{2}\upsilon^{3}}{3h\left(2J+1\right)}\right]\left[X_{ed}S_{ed}+X_{md}S_{md}\right]$$

The average magnetic and electric dipole contributions are denoted by $A_{md}$ and $A_{ed}$, respectively. Expression (24) yields the branching ratio ($\beta_{R}$) for the upper state J.

(24) $$\beta_{R}=\frac{A\left(aJ,bJ^{\prime }\right)}{\left(A_{ed}+A_{md}\right)\left|aJ,bJ^{\prime }\right|}$$

The radiative lifetime (τ) of the emitting state is related to the global spontaneous probabilities $A\left(aJ,bJ^{\prime }\right)$ [19] for the overall transitions.

(25) $$\tau_{r}=\frac{1}{\sum \left(A_{ed}+A_{md}\right)\left|aJ,bJ^{\prime }\right|}$$

The upper state’s total angular momentum is denoted by J.

### 3.5. Spectra of Absorption and Judd–Ofelt Analysis

We provide data for the full RE^3+^ series, including experimental and calculated oscillator strength for the corresponding transitions, absorption spectra, J–O parameters, and root mean square (RMSE) error of the oscillator strength calculations.

#### 3.5.1. Neodymium (Nd^3+^)

**Figure 5:**
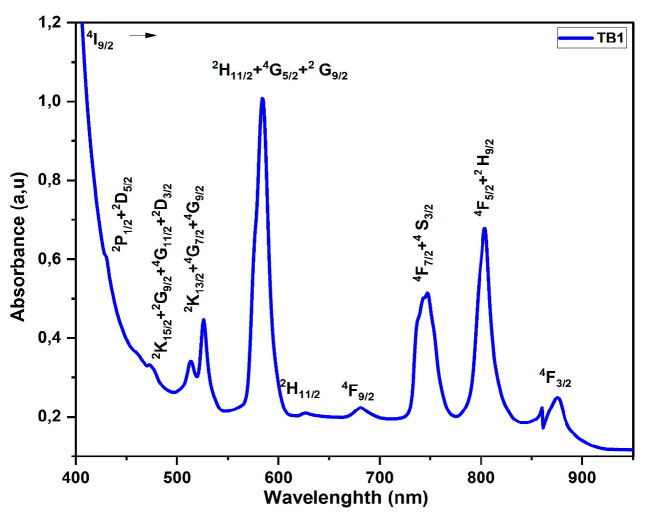
Absorption spectra of Nd^3+^-doped glass.

From 350 to 1200 nm, Figure 5 displays the absorption bands that were produced when Nd^3+^ ions were doped into glass. The spectral lines from the lowest level, ^4^I_9/2_, to the highest, the excited levels, are clearly visible. Table 4 presents the experimental and theoretical oscillator strengths for the transitions from ^4^F_3/2_, ^4^F_5/2_ + ^2^H_9/2_, ^4^F_7/2_ + ^4^S_3/2_, ^4^F_9/2_, ^2^H_11/2_, ^2^H_11/2_ + ^4^G_5/2_ + ^2^G_9/2_, ^2^K_13/2_ + ^4^G_7/2_ + ^4^G_9/2_, ^2^K_15/2_ + ^2^G_9/2_ + ^4^G_11/2_ + ^2^D_3/2_, and ^2^P_1/2_ + ^2^D_5/2_ [20]. The experimental and calculated oscillator strengths for the Nd^3+^ ions agreed with the J–O calculations for the most part, but there were some challenges in determining the oscillator strength in the UV region due to the overlapping bands of the following transitions: ^4^I_9/2_ (^2^P_1/2_), (^2^K_15/2_, ^2^G_9/2_, ^2^D_3/2_, ^2^G_11/2_), and (^2^K_13/2_, ^4^G_7/2_, ^4^G_9/2_). Other authors have suggested that the most effective transition is the hypersensitive ^4^I_9/2_ (^4^G_5/2_, ^2^G_7/2_$\left|∆S\right|\leq 0,\left|∆L\right|,\left|∆J\right|\leq 2)$). These findings follow the same quadrupole selection rules ($\left|∆S\right|\leq 0,\left|∆L\right|,\left|∆J\right|\leq 2)$ as those found in bismuth borated glasses and other fluorophosphate glasses [21,22].

#### 3.5.2. Samarium (Sm^3+^)

**Figure 6:**
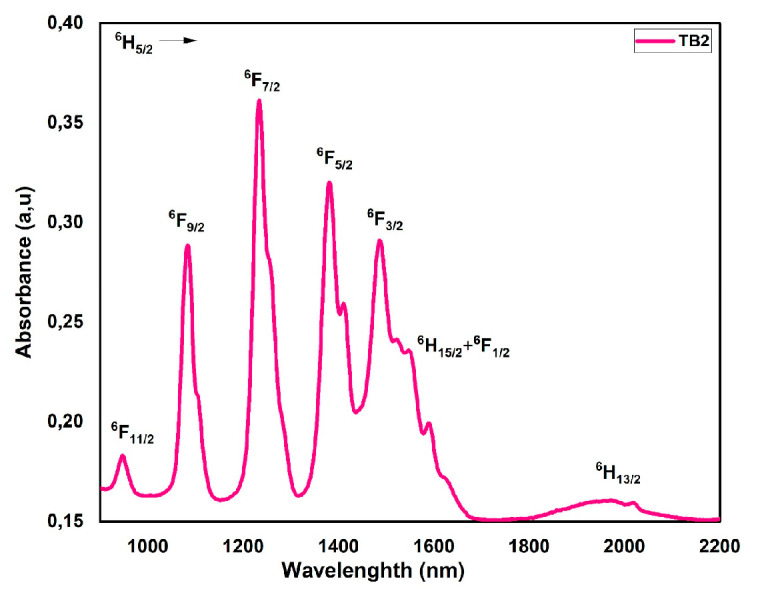
Absorption spectra of Sm^3+^-doped glass.

Visible range absorption spectra were assigned, as seen in Figure 6. Considering the intricacy of the total of the ^2S+1^L_J_ transitions, it was not surprising that calculating the J–O parameters was complex. The oscillator strengths at the ground level ^6^H_5/2_ and the excited levels ^6^H_13/2_, ^6^F_1/2_ + ^6^H_15/2_, ^6^F_3/2_, ^6^F_5/2_, ^6^F_7/2_, ^6^F_9/2_, and ^6^F_11/2_ were measured experimentally and computed [23]. As shown in Table 5, there was a high agreement between the experimental and calculated oscillator strengths and the J–O parameters, proving the accuracy of the J–O predictions. Consistent with previous research by authors such R. Van Deun et al. [18], the findings show that the strongest transitions occur in the near-infrared (NIR) spectrum between 950 nm and 1600 nm.

#### 3.5.3. Dysprosium (Dy^3+^)

**Figure 7:**
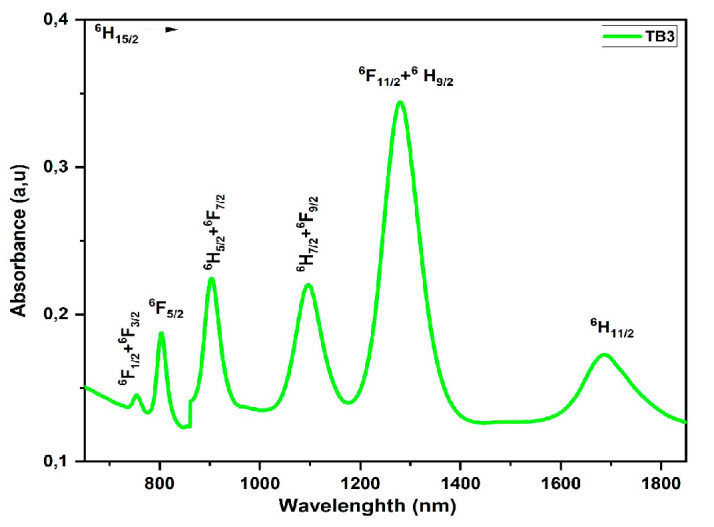
Absorption spectra of Dy^3+^-doped glass.

In Figure 7, we see the spectra of TB3 glass, which has many bands concentrated in the 700 to 1900 nm range. The transition from the ground state ^6^H_15/2_ to the excited states ^6^H_11/2_, ^6^F_11/2_ + ^6^ H_9/2_, ^6^F_9/2_ + ^6^H_7/2_, ^6^F_7/2_ + ^6^H_5/2_, ^6^F_5/2_, and ^6^F_3/2_ + ^6^F_1/2_, respectively, is reflected in absorption bands at 1686, 1279, 1095, 903,803, and 753 nm for Dy^3+^ with the 4f^9^ electronic configuration [24]. Table 6 shows the calculated and experimental oscillator strengths, as well as the J–O parameters.

#### 3.5.4. Erbium (Er^3+^)

**Figure 8:**
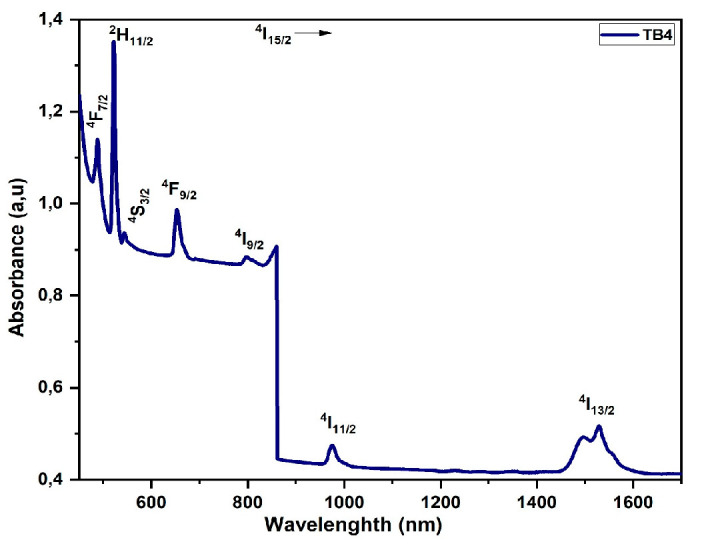
Absorption spectra of Er3^+^-doped glass.

Starting with the ground state ^4^I_15/2_ to the excited states ^4^I_13/2_, ^4^I_11/2_, ^4^I_9/2_,^4^F_9/2_, ^4^S_3/2_, ^2^H_11/2_, and ^4^F_7/2_, the absorption transitions of Er^3+^ ions in tellurite glass connect with absorption bands centered about 1529, 974, 796, 652, 544, 522, and 486 nm [14,25], which are presented in Figure 8. Table 7 shows there is no overlap between the spectra and J–O parametrization. The small RMS confirms that the intensities of the experimental and theoretical oscillators are very similar.

For the lanthanide series, we now know the J–O parameters. Since the intensities of the *f-f* transitions in lanthanides are ligand-dependent, the findings indicate the predicted trends of the (Ω_2_, Ω_4_, Ω_6_) parameters with the rising of *f-f* electrons across the rare-earth series. The RMSE measures how well the data fit the model. In addition, we compared the (Ω_2_, Ω_4_, Ω_6_) calculations in our matrices to those in the scientific literature (Table 8) [8,25,26,27,28,29,30,31,32,33,34,35,36,37,38,39].

The molecular nature of metal–ligand bonds has also been connected to the J–O parameters. Due to the matrix structure complex that these ligands are a part of, the Ω_2_ is dependent on a hypersensitive transition. Due to the fact that f orbitals are far more insulated from the environment than metal d orbitals, consideration must be taken while inferring the spectroscopic and structural connection. On the other hand, the matrix plays an important role, because it provides a broad compositional field that changes with the glass transition, crystallization temperature, and melting temperature. Consequently, these variables affect the dimensions of intensity in different ways. Therefore, the parameters of intensity are still within the experimentally acceptable range [18].

The J–O parameter trend over the lanthanides in tellurite glasses is well recognized. When the number of *f-f* electrons is higher, the Ω_2_ behavior tendency increases, which is similar to other matrices like fluorohafnate glasses and oxyfluoride glass ceramics.

The high oscillator strengths at the hypersensitive transitions may explain why the Ω_2_ values of Nd^3+^ and Dy^3+^ ions are so large relative to the predicted trend. When the values of the individual matrix’s ${\left|U^{2}\right|}^{2}{\left|U^{2}\right|}^{2}$ elements are large, the resulting Ω_2_ parameter are also large. Furthermore, it is difficult to determine the exact values of Ω_2_ due to the overlap of bands in the UV region, as shown in Figure 4 and Figure 6. This is because the observed and theoretical oscillator strengths for Nd^3+^ and Dy^3+^ are consistent. As a consequence, it is possible to derive many transitions from a single, complicated absorption band.

In fact, the predicted and experimental findings for the oscillator strengths of the hypersensitive transitions were more in agreement than those for the lesser oscillator strength of the UV transitions (i.e., Nd^3+^ and Dy^3+^). According to R. Van Deun et al. [8,18], the absorption spectra of Er^3+^ exhibit strong, nonoverlapping absorption bands, equally scattered from the UV area to the NIR region; hence, the Ω_2_ values for these spectra do not cause any complications for J–O calculations. The strength of the oscillator may be calculated, and the J–O parameters can be estimated.

For Sm^3+^, the transition spectra and computed modified J–O parameters exhibited good agreement, with low RMSE and non-negative parameter values for the predicted oscillator strengths.

The findings in Table 8 indicate that the trend towards a series that is suitable with the literature [18] decreases for the parameters Ω_4_ and Ω_6_. The nuclear charge increased by 4f ^N^ causes lanthanide shell contraction, which causes a decrease in the radius integrations in the ${\left|U^{4,6}\right|}^{2}$ matrix elements, an observable decreasing tendency [22].

Covalence of the RE^3+^-F bond was investigated by comparing the findings to those of previous spectral studies published in the literature (Table 8). There was no obvious correlation between the spectral profile and the glass composition in our data. Therefore, it is not obvious that the spectral profile of the hypersensitive transition is dependent on the tellurite concentration, but the data clearly suggest that the intensity of the oscillator increases with the higher concentration.

### 3.6. Raman Spectra

**Figure 9:**
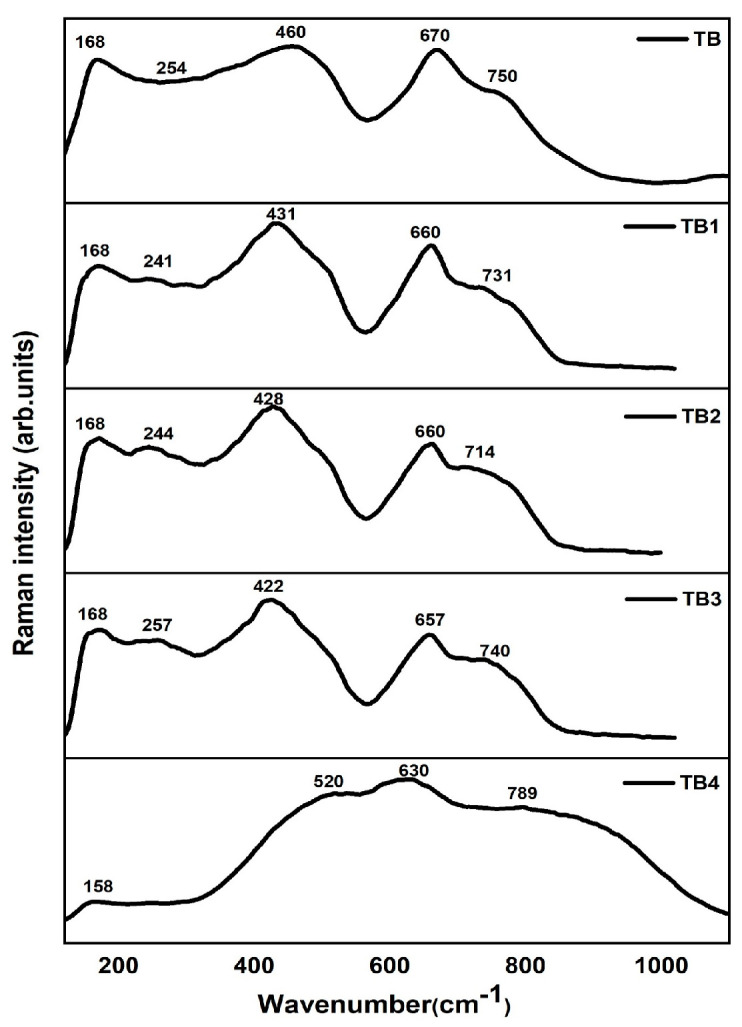
Raman spectra of TB glasses.

The glass samples (80-y) TeO_2_-20 BiCl_3_-y RE_2_O_3_ (y = 0, 0.6% mol; RE = Nd, Sm, Dy, and Er) have Raman spectra that are obtained in the spectral range of 50–1200 cm^−1^ (Figure 9). Table 9 lists the Raman band assignments, while Table 10 lists the observed Raman band positions for all compositions.

**Figure 10:**
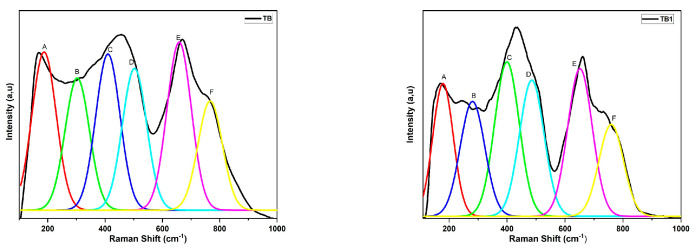

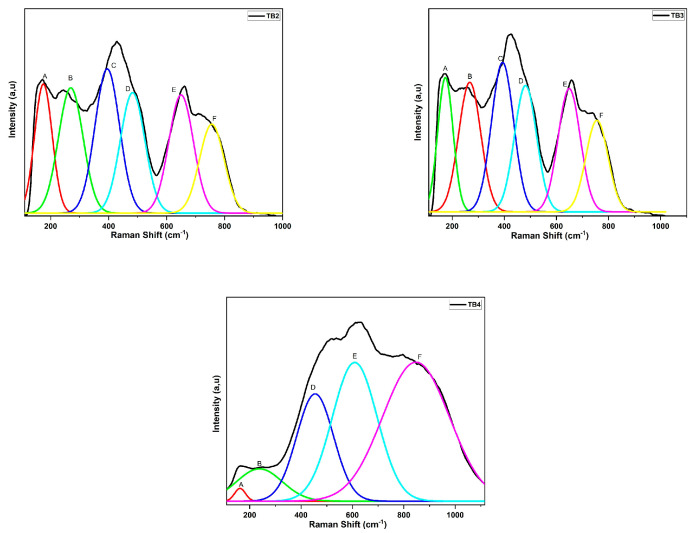
Peak deconvolution of Raman spectra of the (80-y) TeO_2_-20 BiCl_3_-y RE_2_O_3_ (y = 0, 0.6% mol; RE = Nd, Sm, Dy, and Er) glass system.

Figure 10 displays the deconvoluted Raman spectra for TB. Six symmetrical Gaussian peaks can be found in the Raman spectra of the TB glass, which are located around 163–188, 237–303, 394–411, 453–503, 609–658, and 757–854 cm^−1^. Peaks A, B, C, D, E, and F are used to determine them, respectively.

According to several tellurite glasses, including TeO_2_-BiCl_3_ [40], ZnO-Bi_2_O_3_- TeO_2_ [41], TeO_2_-TiO_2_-WO_3_-Nd_2_O_3_ [42], TeO_2_-ZnO-Na_2_CO_3_-Er_2_O_3_ [43], and TeO_2_- TiO_2_-WO_3_-Dy_2_O_3_ [44], the Raman band 394–854 cm^−1^ appears to be characteristic for a tellurite matrix. It is important to note that the TeO_2_ network is made up of a number of asymmetric structural elements, including TeO_4_ trigonal bipyramids (tbp), TeO_3+1_ polyhedra, and TeO_3_ trigonal pyramids (tp). The TeO_4_ group also contains two axial and two equatorial oxygen atoms and doping with rare-earth ions has a significant impact on how TeO_4_ tbp transforms into TeO_3+1_ and TeO_3_ tp units. Additionally, the addition of a network modifier to TeO_2_-based glasses causes the Te-O-Te bonds to break, resulting in the creation of non-bridging oxygen (NBO) atoms and a shift in the Te coordination polyhedron from TeO_4_ to TeO_3_ [3].

The collective modes of local structures and heavy metal ion vibrations, such as the motion of Bi^3+^ or Bi^+3^ cations in BiO_6_ octahedral and/or BiO_3_ pyramidal units, are responsible for the observed band labeled (A) around 163–188 cm^−1^, which was observed when studying the structure of these glasses [5,15]. Bi-O-Bi and Bi-O vibrations of BiO_6_ octahedral units may be responsible for the band designated (B) at 237–303 cm^−1^ [40].

The TeO_4_ axial bending vibration mode (O_ac_-Te-O_ac_) at corner sharing sites, which combines the structure’s equatorial oxygen atoms with axial oxygen atoms, is responsible for the band designated (C) around 394–411 cm^−1^ in the sample. Sample TB4 does not include a peak around 360 cm^−1^, which may be attributed to either TeO_3_ tp or the Er-O bond; therefore, its presence may have been hidden by the strong Raman response of the TeO_2_ matrix or have been very low. Symmetrical or bending vibrations of Teeq-Oax-Te connections at corner sharing sites in TeO_4_ [40,45] account for the band designated (D) at 453–503 cm^−1^. Anti-symmetrical stretching of the continuous TeO_4_ tbps network is responsible for the (E) band seen between 609 and 658 cm^−1^ [2,42]. The (F) band at 757–854 cm^−1^ is due to Te-O and Te=O bond stretching vibrations in non-bridging oxygen in TeO_3_ tps and TeO_3+1_ polyhedra or Te_2_O_7_ bridged tetrahedra (Te-O-Te antisymmetric stretch) [40,42].

## 4. Conclusions

Research on rare-earth ion-doped tellurite glass was performed. It was observed that, when the number of doped ions varied, unbridged oxygen formed, causing a change in density. The glasses we produced had excellent thermal stability (>100 °C), as shown by the thermal inverted. The measurements for the refractive index showed an extremely high value (n = 2.47).

Studies of the Raman spectrum have shown that the addition of rare-earth ions modifies the structure of the system under investigation. We can deduce the following conclusions from this:

TeO_2_ exists in the TeO_4_ trigonal bipyramid, TeO_3_ tps, and TeO_3+1_ polyhedral structural unit with non-bridging oxygen (NBO).

In all of the studied glasses, bismuth acts as both network-forming BiO3 pyramidal units and network-changing BiO_6_ octahedral units.

The presence of rare-earth ions influences the formation of distinct tellurite structural units, thus the appearance of shifting Raman bands toward lower or higher wave numbers with varying intensities.

Trivalent lanthanide ions in tellurite glasses have been characterized by their J–O parameters, which we report. In the cases of Ω_2_ and Ω_4_, the findings show that there is no consistent pattern in the abundance of 4f electrons. However, Ω_6_ provides a universal connection that holds true not just in tellurite glass containers but also in other host matrices.

## Figures and Tables

**Table 1:** The nominal composition of the (80-y) TeO_2_-20 BiCl_3_-y RE_2_O_3_ (y = 0, 0.6% mol; RE = Nd, Sm, Dy, and Er) glass sample codes.

Sample Codes	Composition
TB	80 TeO_2_-20 BiCl_3_
TB1	79.4 TeO_2_-20 BiCl_3_-0.6 Nd_2_O_3_
TB2	79.4 TeO_2_-20 BiCl_3_-0.6 Sm_2_O_3_
TB3	79.4 TeO_2_-20 BiCl_3_-0.6 Dy_2_O_3_
TB4	79.4 TeO_2_-20 BiCl_3_-0.6 Er_2_O_3_

**Table 2:** The glass transition temperature T_g_, onset crystallization temperatures T_x_, crystallization temperatures T_p_, thermal stability factor ΔT, Hruby parameter, and glass stability range S of the (80-y) TeO_2_-20 BiCl_3_-y RE_2_O_3_ (y = 0, 0.6% mol; RE = Nd, Sm, Dy, and Er) glass system.

Sample Code	T_g_ (±1 °C)	T_x1_ (±1 °C)	T_p1_ (±1 °C)	T_x2_ (±1 °C)	ΔT (±1 °C)	H	S
TB	296	412	-	-	116	0.39	-
TB1	286	390	433	455	104/169	0.36/0.59	15.63/1.18
TB2	285	394	455	-	109	0.38	23.32
TB3	333	429	477	-	106	0.32	15.27
TB4	286	413	430	435	127/149	0.44/0.52	7.54/10.41

**Table 3:** Physical and optical glass system parameters (80-y) TeO_2_-20 BiCl_3_-y RE_2_O_3_ (y = 0, 0.6% mol; RE = Nd, Sm, Dy, and Er).

Sample Code	TB	TB1	TB2	TB3	TB4
Density ρ_g_ (g/cm^3^)	5.506	5.475	5.468	5.482	5.488
M_wt_ (g/mol)	190.746	191.879	191.953	192.098	192.156
Molar Volume V_mol_ (cm^3^/mol)	34.65	35.05	35.10	35.04	35.01
Oxygen Volume V_OXG_ (cm^3^/mol)	46.185	45.825	45.749	45.831	45.868
OPD (g atom/l)	21.65	21.83	21.87	21.83	21.81
Refraction loss (R_Loss_)	0.168	0.169	0.171	0.175	0.179
Dielectric constant (ε)	5.71	5.76	5.81	5.95	6.10
Molar Refractivity R_mol_ (cm^3^/mol)	21.171	21.499	21.614	21.823	22.044
Molar Polarizability α_mol_ (A°)^3^	8.401	8.531	8.576	8.601	8.748
$E_{opt}^{ind}E_{opt}^{ind}$(eV)	2.99	2.98	2.92	2.84	2.75
Refractive index (n)	2.39	2.40	2.41	2.44	2.47
Polaron radius r_pl_ (A°)	0	8.60	8.61	8.60	8.60
Inter ionic distance r_in_ (A°)	0	21.35	21.37	21.35	21.35
Field strength Fs (×10^14^ cm^−2^)	0	4.06	4.05	4.06	4.06
RE ion concentration N_RE_ (×10^20^ ions/cm^3^)	0	1.031	1.029	1.031	1.032

**Table 4:** The oscillator strengths and J–O parameters determined from the absorption spectra of TB1 glass.

^4^I_9/2_ 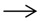	Λ (nm)	Oscillator Line Strength × 10^−6^	
		f_exp_	f_cal_
^4^F_3/2_	876	3.56	3.72
^4^F_5/2_ + ^2^H_9/2_	802	10.92	10.50
^4^F_7/2_ + ^4^S _3/2_	744	9.92	10.16
^4^F_9/2_	682	0.88	0.95
^2^H_11/2_ + ^4^G_7/2_ + ^2^G_7/2_	584	30.11	30.14
^2^K_13/2_ + ^4^G_7/2_ + ^4^G_9/2_	526	9.04	8.69
^2^K_15/2_ + ^2^G_9/2_ + ^4^G_11/2_ + ^2^D_3/2_	473	0.82	1.99
^2^P_1/2_ + ^2^D_5/2_	430	0.67	1.52
Ω_2_ = 3.34 × 10^−20^ cm^2^, Ω_4_ = 3.86 × 10^−20^ cm^2^, Ω_6_ = 3.54 × 10^−20^ cm^2^ RMSE = 0.313 × 10^−6^			

**Table 5:** The oscillator strengths and J–O parameters determined from TB2 glass absorption spectra.

^6^H_5/2_ 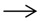	Λ (nm)	Oscillator Line Strength × 10^−6^	
		f_exp_	f_cal_
^6^H_13/2_	1970	0.35	0.32
^6^F _½_ + ^6^HI_15/2_	1590	0.64	0.28
^6^F_3/2_	1488	0.70	1.20
^6^F_5/2_	1382	2.45	2.30
^6^F_7/2_	1234	4.80	4.65
^6^F_9/2_	1084	3.09	3.26
^6^F_11/2_	948	0.45	0.53
Ω_2_ = 0.48 × 10^−20^ cm^2^, Ω_4_ = 2.04 × 10^−20^ cm^2^, Ω_6_ = 1.83 × 10^−20^ cm^2^ RMSE = 0.169 × 10^−6^			

**Table 6:** The oscillator strengths and J–O parameters determined from TB3 glass absorption spectra.

^6^H_15/2_ 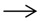	Λ (nm)	Oscillator Line Strength × 10^−6^	
		f_exp_	f_cal_
^6^H_11/2_	1687	1.69	2.24
^6^F_11/2_ + ^6^ H_9/2_	1279	9.28	9.24
^6^F_9/2_ + ^6^ H_7/2_	1097	3.58	3.75
^6^F_7/2_ + ^6^ H_5/2_	903	4.23	3.74
^6^F_5/2_	803	1.77	1.92
^6^F_3/2_ + ^6^F_1/2_	754	0.26	0.36
Ω_2_ = 4.91 × 10^−20^ cm^2^, Ω_4_ = 0.63 × 10^−20^ cm^2^, Ω_6_ = 2.29 × 10^−20^ cm^2^ RMSE = 0.260 × 10^−6^			

**Table 7:** The oscillator strengths and J–O parameters determined from TB4 glass absorption spectra.

^4^I_15/2_ 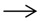	Λ (nm)	Oscillator Line Strength × 10^−6^	
		f_exp_	f_cal_
^4^I_13/2_	1529	2.63	3.10
^4^I_11/2_	974	0.74	1.45
^4^I _9/2_	796	0.32	0.35
^4^F_9/2_	652	3.05	3.38
^4^S_3/2_	544	0.48	1.26
^2^H_11/2_	522	10.93	10.92
^4^F_7/2_	486	5.40	4.42
Ω_2_ = 3.38 × 10^−20^ cm^2^, Ω_4_ = 0.72 × 10^−20^ cm^2^, Ω_6_ = 1.55 × 10^−20^ cm^2^ RMSE = 0.3866 × 10^−6^			

**Table 8:** Comparison of the Judd–Ofelt parameters Ω_λ_ (λ = 2; 4; 6) of glass sample (80-y) TeO_2_-20 BiCl_3_-y RE_2_O_3_ (y = 0, 0.6% mol; RE = Nd, Sm, Dy, and Er).

RE^3+^	Glass Matrix	Ω_2_	Ω_4_	Ω_6_	Ωλ Tendency	Reference
Nd^3+^	TB1	3.34	3.86	3.54	Ω_2_ > Ω_6_ > Ω_4_	Present Work
	TLF	5.61	4.17	5.44	Ω_2_ > Ω_6_ > Ω_4_	[25]
	TBZBNd0.5	2.89	1.35	1.59	Ω_2_ > Ω_6_ > Ω_4_	[26]
	TW-1 Nd	4.60	3.30	3.50	Ω_2_ > Ω_6_ > Ω_4_	[27]
	ZNNd0.5	3.76	1.16	3.23	Ω_2_ > Ω_6_ > Ω_4_	[28]
Sm^3+^	TB2	0.40	2.16	1.19	Ω_4_ > Ω_6_ > Ω_2_	Present Work
	LBZnFSm	0.4117	9.46	8.92	Ω_4_ > Ω_6_ > Ω_2_	[29]
	PKAMZFSm	0.41	2.99	2.67	Ω_4_ > Ω_6_ > Ω_2_	[30]
	BLNS	3.92	8.17	5.82	Ω_4_ > Ω_6_ > Ω_2_	[31]
	LBTAF	0.27	2.52	2.47	Ω_4_ > Ω_6_ > Ω_2_	[32]
Dy^3+^	TB3	4.91	0.63	2.29	Ω_2_ > Ω_6_ > Ω_4_	Present Work
	SFBiBDy1	39.52	6.42	9.15	Ω_2_ > Ω_6_ > Ω_4_	[33]
	Lead borate	4.90	0.94	2.07	Ω_2_ > Ω_6_ > Ω_4_	[34]
	TPA	6.45	1.27	2.90	Ω_2_ > Ω_6_ > Ω_4_	[8]
	BSLNAD3	1.29	0.35	0.42	Ω_2_ > Ω_6_ > Ω_4_	[35]
Er^3+^	TB4	3.38	0.72	1.55	Ω_2_ > Ω_6_ > Ω_4_	Present Work
	0.5ErBT	5.73	2.01	2.37	Ω_2_ > Ω_6_ > Ω_4_	[36]
	BTNMES	3.53	1.18	2.06	Ω_2_ > Ω_6_ > Ω_4_	[37]
	TZNBG2	5.02	0.95	1.21	Ω_2_ > Ω_6_ > Ω_4_	[38]
	TPBKZFEr05	7.23	1.02	1.46	Ω_2_ > Ω_6_ > Ω_4_	[39]

**Table 9:** Peak positions in cm^−1^ of the prepared glasses.

Sample Code	Peak Position in cm^−1^					
	**A**	**B**	**C**	**D**	**E**	**F**
TB	188	303	411	503	658	767
TB1	181	280	398	485	653	761
TB2	175	270	394	482	648	757
TB3	175	266	395	481	650	758
TB4	163	237	Not appeared	453	609	854

**Table 10:** Assignments of deconvoluted Raman bands of the TB glass samples.

Band Position	Peak	Assignments
163–188 cm^−1^	A	Heavy metal bonds.
237–303 cm^−1^	B	Bending vibrations of Bi–O–Bi or O–Bi–O linkages of BiO_6_.
394–411 cm^−1^	C	Bending vibrations of O-Te-O bonds in TeO_4_ units.
453–503 cm^−1^	D	Symmetric stretching vibrations of the continuous network composed of TeO_4_ tbp.
609–658 cm^−1^	E	Antisymmetric stretching vibrations of Te-O-Te bridge bonds occurring in TeO_4_ tbp units and BiO_6_ or BiCl_6_ octahedral units.
757–854 cm^−1^	F	Stretching vibration of TeO_3_ units. Stretching vibrations of bonds between Te and non-bridged oxygen atoms occurring in TeO_3+1_ units.

## Data Availability

Not applicable.
